# Patterns of Screen Time Among Rural Mexican-American Children on the New Mexico-Mexico Border

**DOI:** 10.5888/pcd15.180070

**Published:** 2018-09-13

**Authors:** Jill A. McDonald, Christopher Sroka, Elizabeth Olivares, Merranda Marin, Maria Gurrola, Joseph R. Sharkey

**Affiliations:** 1 Department of Public Health Sciences, College of Health and Social Services, New Mexico State University, Las Cruces, New Mexico; 2 Southwest Institute for Health Disparities Research, College of Health and Social Services, New Mexico State University, Las Cruces, New Mexico; 3 Economics, Applied Statistics and International Business Department, College of Business, New Mexico State University, Las Cruces, New Mexico; 4 Department of Family and Child Science, College of Agricultural, Consumer and Environmental Sciences, New Mexico State University, Las Cruces, New Mexico; 5 School of Social Work, New Mexico State University, Las Cruces, New Mexico; 6 Department of Health Promotion and Community Health Sciences, Texas A&M School of Public Health, College Station, Texas

## Abstract

**Introduction:**

The prevalence of obesity is 26% among Hispanic children and teenagers and 47% among Hispanic adults. One contributor to obesity is sedentary behavior, such as using electronic screen devices (ie, screens). Low-income and Hispanic youths spend more time using such devices than other youths.

**Methods:**

We interviewed 202 parents of Mexican-origin children aged 6 to 10 years in 2 rural communities near the US–Mexico border to determine screen use among children. We tested for associations between covariates and heavy screen use (≥4 hours/day) and calculated adjusted odds ratios (AORs) to identify independent, modifiable risk factors for such use.

**Results:**

More than two-thirds (68.3%) of households had an annual income of less than $24,000, 89.1% spoke primarily Spanish, and 92.1% had internet access. The percentage of children with heavy screen use was 14.9% on weekdays and 25.2% on weekends. Smartphones were used by 62.4% of children, desktops or laptops by 60.9%; homework was the most common reason for use of these devices. One in 3 children used them for social media. Increased odds of heavy screen use were associated with having a television on while the child ate (weekday AOR = 3.02; 95% confidence interval [CI], 1.08–8.45 and weekend AOR = 2.38; 95% CI, 1.04–5.40) and using electronics to entertain (weekend AOR = 2.94; 95% CI, 1.15–7.51). More than 3 family meals per week (AOR = 0.40; 95% CI, 0.17–0.94 compared with ≤3 meals) and 2 or 3 family activities per week (AOR = 0.33; 95% CI, 0.12–0.87 compared with ≤1 activity) were associated with decreased odds of heavy weekend use.

**Conclusion:**

Even in low-income, Spanish-speaking communities, children have access to electronic devices, social media, and the internet, and a substantial fraction of them are heavy users. Efforts to reduce screen time might focus on understanding and changing the social norms that promote it.

## Introduction

In 2015–2016, Hispanic adults had a higher age-adjusted rate of obesity (47.0%) than non-Hispanic white (37.9%) or non-Hispanic black (46.8%) adults in the United States (1). Moreover, the prevalence of obesity in 2015–2016 among children and teenagers aged 2 to 19 years was 25.8% among Hispanics, 22.0% among non-Hispanic blacks, and 14.2% among non-Hispanic whites (1). The 2015 Youth Risk Behavior Survey showed that 16.2% of Hispanic and 13.3% of non-Hispanic white ninth-graders in New Mexico were obese, while an additional 16.8% of Hispanic and 15.5% of non-Hispanic white ninth-graders were overweight. Obesity rates in both populations are increasing (2).

Behaviors that contribute to obesity among children and teenagers include sedentary behavior and the consumption of excessive calories (3–5). Sedentary behavior is defined as any waking behavior that has a low level of energy expenditure (<1.5 metabolic equivalents) while in a sitting, reclining, or lying posture (6). The component of such behavior that is studied most often is screen time. Screen time is time spent on screen-based behaviors (6), such as watching television, playing video games, and using computers, smartphones, or other electronic devices with screens. Use of devices with screens other than televisions has increased dramatically in the United States in recent years (7).

Perhaps one reason the rate of obesity is higher among Hispanic children and teenagers than among their non-Hispanic counterparts is that the former spend more time using electronic screen devices (8). For example, in the 2015 New Mexico Youth Risk Behavior Survey, 27.3% of Hispanic and 21.7% of non-Hispanic white ninth-graders spent 3 hours or more watching television each weekday (2). And in the 2015 New Mexico Youth Risk and Resilience Survey in the largely Hispanic county of Otero, in the US–Mexico border region, 27.7% of middle-school students (sixth- to eighth-graders) watched 3 hours or more of television, and 28.5% used computers or video games for 3 hours or more on weekdays (9). Consistently, low-income and racial/ethnic minority children and teenagers report more time using electronic devices for recreational purposes than do their non-Hispanic white counterparts (8). Other demographic groups associated with greater screen time include boys, older children, younger mothers, and less-educated parents (7,8,10). Little is known about screen time in Hispanic subpopulations, especially Hispanic children in elementary school. Studying screen time in younger children is important because risk factors for obesity can begin to operate as early as infancy (11).

The *Salud Para Usted y Su Familia* (Health for You and Your Family) project (12) is studying the determinants of obesity among Mexican American children in rural, low-income border communities in New Mexico. As a first step in designing an intervention to reduce the risk for childhood obesity in these communities, we collected data on the prevalence of risk factors, including screen time. The main objective of this study was to describe the demographic correlates of heavy screen use among Mexican American children in 2 small, rural communities on the New Mexico–Mexico border. A secondary objective was to assess the association of selected modifiable household norms with heavy screen use.

## Methods

From July through December 2016, we conducted a cross-sectional survey of 202 mothers or primary caregivers of Mexican-origin children aged 6 to 10 years (in grades kindergarten through 4) in 2 *colonias* (rural communities that lack adequate water, sewer, or decent housing) (13). We recruited study participants from the unincorporated community of Chaparral (population, 14,631) in Otero County and Doña Ana County and the village of Columbus (population, 1,244) in Luna County (14). Chaparral and Columbus are 20 and 3 miles from the Mexican border and 84% and 88% Hispanic, respectively (14).

We hired and trained *promotores de salud* (promoters of health), bilingual indigenous community health workers, as promotor–researchers to recruit participants and collect data for the project (15,16). Promotor–researchers recruited a convenience sample from their communities by approaching potential respondents door-to-door and at schools, school bus stops, shopping centers, and community events. When 2 parents were available, mothers were preferred as participants. Promotor–researchers determined eligibility by administering a 9-item questionnaire. Eligibility criteria included having lived in the community for at least 1 year, being the primary caregiver for a Mexican American child aged 6 to 10 years, and living with a spouse or partner who shared childcare responsibilities. Mexican origin was defined as Mexican nativity in the child or 1 or more of the child’s biological parents or grandparents.

Our goal was a sample size of 200, 100 from each community. Promotor–researchers approached 1,093 individuals, of whom 1,091 (99.8%) completed the questionnaire. Among these, 260 (23.8%) were eligible. The modal reason for ineligibility was not having a child aged 6 to 10 years. Among eligible respondents, 202 (77.7%) signed informed consent agreements, and all those who signed completed interviews.

Promotor–researchers administered the informed consent and the study instruments in English or Spanish, depending on the respondent’s preference. The primary study instrument, an 88-item survey, took 45 minutes and was conducted at the time of recruitment or later at a convenient location. Study participants received a $5.00 gift card.

We asked participants with 2 or more children aged 6 to 10 years to choose 1 child and answer survey questions with that 1 reference child in mind. Interviewers prompted participants to respond about that child with phrases such as, “Going back to the child you were thinking about . . ..”

### Variables

The 88 survey questions covered a range of factors associated in the literature with childhood obesity, including demographic variables, diet, and physical activity. It also included factors associated with screen time: 1) internet access; use of smartphones, computers or laptops, and other electronic devices among children, mothers, and fathers; 2) household norms related to screen use (Box); and 3) reasons for use and types of electronic devices used by all children in the household.

**Box. Household Norms Related to Screen Use as Defined by the Questions Below.**

• Is the TV on when your child eats?

• When eating together as a family, is there anyone who uses electronics (cell phone, games, etc.)?

• During a normal week, how often does your family eat a meal together?

• When your child misbehaves, do you ever take away his/her outdoor play time?

• When your child misbehaves, do you ever take away his/her electronics?

• Does it ever seem the only way to keep your child entertained is to encourage his/her use of TV, tablet, video games, or other electronics?

• How many times a week does your family do active things together?

We defined screen time as the number of hours per day that the child used electronic screen devices at home. Mothers were asked, “How many **hours** does your child spend at home on a normal day **during the week** using electronics (TV, videogames, computer games, cell phone)?” and “How many **hours** does your child spend at home on a normal day **on the weekend** using electronics (TV, videogames, computer games, cell phone)?” Possible responses were none, 1 or 2 hours, 3 hours, or 4 hours or more. The 2 outcome variables were heavy screen use on weekdays and heavy screen use on weekends. We defined heavy screen use as 4 hours or more per day (17).

### Analysis

To assess which variables should be included in a multivariate analysis, we first conducted individual tests of association between the outcome variables and potential risk factors. We used χ^2^ and Fisher exact tests for unordered categorical variables and Cochran–Armitage tests for trend for ordered variables. Variables were included in the multivariate model if 1) the variable was associated (*P* < .25) in the weekday or weekend analysis, or 2) the variable was associated with screen time in the literature (ie, child’s age, child’s sex, maternal education, and income/Medicaid status). Internet access met the first criterion, but it was excluded because none of the heavy users lacked internet access. Weekday and weekend use were fit by using separate models.

Analysis was conducted by using SAS version 9.4 (SAS Institute Inc). The institutional review boards of the institutions with which the authors are affiliated reviewed and approved the study protocol.

## Results

Among the 202 children in the study, 117 (57.9%) were aged 6 to 8 and 85 (42.1%) were aged 9 or 10 (Table 1). Mean age was 8.1 years (standard deviation, 1.4 y). Among the parent respondents, 192 (95.0%) were female, 144 (71.3%) were born in Mexico, 181 (89.6%) had a high school education or less, and 143 (70.8%) had 5 or more members in their household. Among the 202 study households, 99 of 145 (68.3%) had a total monthly income of less than $2,000 (excluding “don’t know” responses); 180 (89.1%) had a member who receives Medicaid, and 180 (89.1%) spoke primarily Spanish. The children used Spanish-language electronic devices exclusively in 46 (22.8%) households; most used English exclusively or English and Spanish. Most (n = 108 [53.5%]) households had cell phone plans, and 92.1% had internet access.

**Table 1:** Characteristics of Study Population and Their Association With Heavy Screen Use (≥4 Screen-Time Hours per Day) on Weekdays and Weekends Among Mexican-Origin Children Aged 6 to 10 Years, Chaparral and Columbus, New Mexico, 2016^a^

Characteristic	Overall, No. (%)^b^ (n = 202)	Weekday (n = 30)		Weekend Day (n = 51)	
		No. (%)	*P* Value	No. (%)	*P* Value
**Child’s age, y**					
6–8	117 (57.9)	15 (12.8)	.34^c^	27 (23.1)	.40^c^
9 or 10	85 (42.1)	15 (17.6)		24 (28.2)	
Mean	8.1	8.1	—	8.3	—
**Child’s sex**					
Male	108 (53.5)	16 (14.8)	>.99^c^	31 (28.7)	.26^c^
Female	94 (46.5)	14 (14.9)		20 (21.3)	
**Maternal age, y^d^**					
20–29	51 (25.6)	10 (19.6)	.25^e^	15 (29.4)	.44^e^
30–39	95 (47.7)	14 (14.7)		17 (17.9)	
≥40	53 (26.6)	6 (11.3)		18 (34.0)	
Mean	35.7	34.2	—	37.5	—
**Maternal birth country**					
United States	58 (28.7)	8 (13.8)	>.99^c^	14 (24.1)	.86^c^
Mexico	144 (71.3)	22 (15.3)		37 (25.7)	
**No. of years of maternal education**					
1–8	45 (22.3)	7 (15.6)	.71^e^	15 (33.3)	.14^e^
9–12	136 (67.3)	21 (15.4)		32 (23.5)	
>12	21 (10.4)	2 (9.5)		4 (19.0)	
**No. of household members**					
3	20 (9.9)	6 (30.0)	.051^e^	11 (55.0)	.002^e^
4	39 (19.3)	6 (15.4)		11 (28.2)	
5	74 (36.6)	11 (14.9)		17 (23.0)	
≥6	69 (34.2)	7 (10.1)		12 (17.4)	
**Monthly household income, $**					
<1,000	41 (20.3)	9 (22.0)	.13^e^	12 (29.3)	.08^e^
1,000–1,999	58 (28.7)	9 (15.5)		14 (24.1)	
2,000–2,999	26 (12.9)	4 (15.4)		3 (11.5)	
≥3,000	20 (9.9)	1 (5.0)		3 (15.0)	
Don’t know	57 (28.2)	7 (12.3)		19 (33.3)	
**Household member receives Medicaid**					
No	22 (10.9)	3 (13.6)	>.99^c^	8 (36.4)	.20^c^
Yes	180 (89.1)	27 (15.0)		43 (23.9)	
**Primary language in household**					
Spanish	180 (89.1)	26 (14.4)	.75^c^	45 (25.0)	.80^c^
English	22 (10.9)	4 (18.2)		6 (27.3)	
**Language child uses for electronics**					
Spanish exclusively	46 (22.8)	9 (19.6)	.59^c^	15 (32.6)	.36^c^
English exclusively	84 (41.6)	11 (13.1)		21 (25.0)	
Both	72 (35.6)	10 (13.9)		15 (20.8)	
**Internet access type**					
Cell phone subscription	87 (43.1)	14 (16.1)	.21^c^	22 (25.3)	.95^c^
DSL/cable subscription	49 (24.3)	6 (12.2)		15 (30.6)	
Both cell phone and DSL/cable subscription	21 (10.4)	3 (14.3)		5 (23.8)	
Other type of internet subscription	15 (7.4)	5 (33.3)		3 (20.0)	
Access to internet without subscription	14 (6.9)	2 (14.3)		3 (21.4)	
No internet access	16 (7.9)	0		3 (18.8)	
**Community of residence**					
Chaparral	102 (50.5)	16 (15.7)	.84^c^	19 (18.6)	.04^c^
Columbus	100 (49.5)	14 (14.0)		32 (32.0)	
**Total**	202 (100.0)	30 (14.9)	—	51 (25.2)	—

a Data collected from 88-item survey of 202 mothers or primary caregivers from July through December 2016. Participants with 2 or more children aged 6 to 10 years were asked to choose 1 child and answer survey questions with that 1 reference child in mind.

b Percentages may not sum to 100 because of rounding.

c Determined by Fisher exact test.

d Values do not sum to 202 because 3 respondents did not answer question.

e Determined by Cochran–Armitage 2-sided trend test.

Approximately one-quarter (53 of 202; 26.2%) of children used screens for more than 2 hours per day during the week at home, and 30 (14.9%) were heavy weekday users. On weekends, 84 (41.6%) children used screens for more than 2 hours per day, and 51 (25.2%) were heavy weekend users. Screen time was greater on weekend days (*P* = .002). Heavy use during weekdays or weekends was not significantly associated with child’s age, child’s sex, or any other demographic characteristic except household size (Table 1). We found a trend toward less screen use on weekends as household size increased. We also found that a greater percentage of children in Columbus (32.0%) than in Chaparral (18.6%) were heavy weekend users.

The unadjusted analysis of 7 household norms (Table 2) found that norms encouraging screen use were common. Six of 7 norms qualified for inclusion in the adjusted analysis, but we included all 7 norms. In the adjusted analysis (Table 3), no demographic variables other than household size were associated with screen time. Larger households were less likely to report heavy weekend screen use. In contrast, 4 of 7 norms were associated with heavy weekday use, heavy weekend use, or both. Most (59.9%) families had the television on while the child ate, a practice associated with heavy screen use both on weekdays (AOR = 3.02; 95% confidence interval [CI], 1.08–8.45) and weekends (AOR = 2.38; 95% CI, 1.04–5.40) (Table 3). Three of 4 families ate meals together more than 3 times per week, and heavy weekend screen use among children in these families was less prevalent than in families who ate meals together 3 times or fewer per week (AOR = 0.40; 95% CI, 0.17–0.94). Parents who used television or electronics for entertaining their child reported heavy weekend use more than twice as often (AOR = 2.94; 95% CI, 1.15–7.51) as parents who did not. Finally, families who were physically active together 2 or 3 times per week were associated with less weekend screen time than families active together at most 1 time (0 or 1) per week (AOR = 0.33; 95% CI, 0.12–0.87).

**Table 2:** Household Norms and Their Association With Heavy Screen Use (≥4 Screen-Time Hours per Day) on Weekdays and Weekends Among Mexican-Origin Children Aged 6 to 10 Years, Chaparral and Columbus, New Mexico, 2016^a^

Household Norm	Category	Overall, No. (%) (n = 202)	Weekday (n = 30)		Weekend Day (n = 51)	
			No. (%)	*P* Value^b^	No. (%)	*P* Value^b^
Is the TV on when your child eats?	No	81 (40.1)	6 (7.4)	.02	13 (16.0)	.01
	Yes	121 (59.9)	24 (19.8)		38 (31.4)	
When eating together as a family, is there anyone who uses electronics (cell phone, games, etc.)?	No	153 (75.7)	20 (13.1)	.25	37 (24.2)	.57
	Yes	49 (24.3)	10 (20.4)		14 (28.6)	
During a normal week, how often does your family eat a meal together?	≤1	18 (8.9)	2 (11.1)	.03	5 (27.8)	.08
	2 or 3	32 (15.8)	10 (31.3)		13 (40.6)	
	>3	152 (75.2)	18 (11.8)		33 (21.7)	
When your child misbehaves, do you ever take away his/her outdoor play time?	No	108 (53.5)	14 (13,0)	.44	28 (25.9)	.87
	Yes	94 (46.5)	16 (17.0)		23 (24.5)	
When your child misbehaves, do you ever take away his/her electronics?	No	17 (8.4)	2 (11.8)	>.99	1 (5.9)	.08
	Yes	185 (91.6)	28 (15.1)		50 (27.0)	
Does it ever seem the only way to keep your child entertained is to encourage his/her use of TV, tablet, video games, or other electronics?	No	172 (85.1)	22 (12.8)	.09	37 (21.5)	.006
	Yes	30 (14.9)	8 (26.7)		14 (46.7)	
How many times a week does your family do active things together?	≤1	80 (39.6)	15 (18.8)	.48	29 (36.3)	.01
	2 or 3	65 (32.2)	8 (12.3)		10 (15.4)	
	>3	57 (28.2)	7 (12.3)		12 (21.1)	
Total	—	202 (100.0)	30 (14.9)	—	51 (25.2)	—

a Data collected from 88-item survey of 202 mothers or primary caregivers from July through December 2016. Participants with 2 or more children aged 6 to 10 years were asked to choose 1 child and answer survey questions with that 1 reference child in mind.

b Determined by Fisher exact test.

**Table 3:** Adjusted Odds Ratios for Associations of Demographic Characteristics and Household Norms With Heavy Screen Use (≥4 Screen-Time Hours per Day) on Weekdays and Weekends Among Mexican-Origin Children Aged 6 to 10 Years, Chaparral and Columbus, New Mexico, 2016^a^

Demographic Variable or Household Norm	Adjusted Odds Ratio (95% Confidence Interval)	
	Weekday	Weekend Day
**Community of residence**		
Chaparral	1 [Reference]	1 [Reference]
Columbus	0.63 (0.24–1.63)	1.22 (0.56–2.65)
**Child’s age**	1.17 (0.83–1.64)	1.24 (0.92–1.66)
**Child’s sex**		
Male	1 [Reference]	1 [Reference]
Female	1.01 (0.41–2.48)	0.63 (0.29–1.38)
**Maternal age**	0.95 (0.89–1.02)	1.04 (0.99–1.10)
**Years of maternal education**	0.96 (0.80–1.16)	1.02 (0.88–1.17)
**No. of household members**	0.79 (0.57–1.10)	0.73 (0.55–0.98)
**Monthly household income, $^b^**		
<1,000	1 [Reference]	—
Don’t know	0.67 (0.18–2.51)	—
1,000–1,999	0.92 (0.28–2.98)	—
≥2,000	0.44 (0.11–1.69)	—
**Household member receives Medicaid^b^**		
No	—	1 [Reference]
Yes	—	0.33 (0.10–1.03)
**Television on during meals^c^**		
No	1 [Reference]	1 [Reference]
Yes	3.02 (1.08–8.45)	2.38 (1.04–5.40)
**Someone uses electronics while eating^c^**		
No	1 [Reference]	1 [Reference]
Yes	1.32 (0.50–3.54)	1.18 (0.49–2.88)
**No. of meals eaten together during the week^c^**		
≤3	1 [Reference]	1 [Reference]
>3	0.44 (0.17–1.16)	0.40 (0.17–0.94)
**Outdoor play time limited for misbehavior^c^**		
No	1 [Reference]	1 [Reference]
Yes	1.25 (0.51–3.05)	0.85 (0.39–1.84)
**Use of electronic devices limited for misbehavior^c^**		
No	1 [Reference]	1 [Reference]
Yes	0.78 (0.14–4.23)	5.84 (0.64–53.05)
**Feels electronics are the only way to keep children entertained^c^**		
No	1 [Reference]	1 [Reference]
Yes	2.17 (0.75–6.30)	2.94 (1.15–7.51)
**No. of times per week family does active things together^c^**		
≤1	1 [Reference]	1 [Reference]
2 or 3	0.58 (0.19–1.75)	0.33 (0.12–0.87)
>3	1.14 (0.36–3.63)	0.96 (0.38–2.46)

a Data collected from 88-item survey of 202 mothers or primary caregivers from July through December 2016. Participants with 2 or more children aged 6 to 10 years were asked to choose 1 child and answer survey questions with that 1 reference child in mind.

b Medicaid participation was used as a proxy for income in the weekend model because 28.2% of participants responded “don’t know” to the income question. For the weekday model, only 3 reference children were heavy users and were not in a Medicaid household, so we chose to include household income in this model, treating the “don’t knows” as a separate category and combining the $2,000-$2,999 and ≥$3,000 groups. Internet access was not included in the models because none of the heavy users were without internet access.

c Household norms were rephrased for this table.

**Figure 1:**
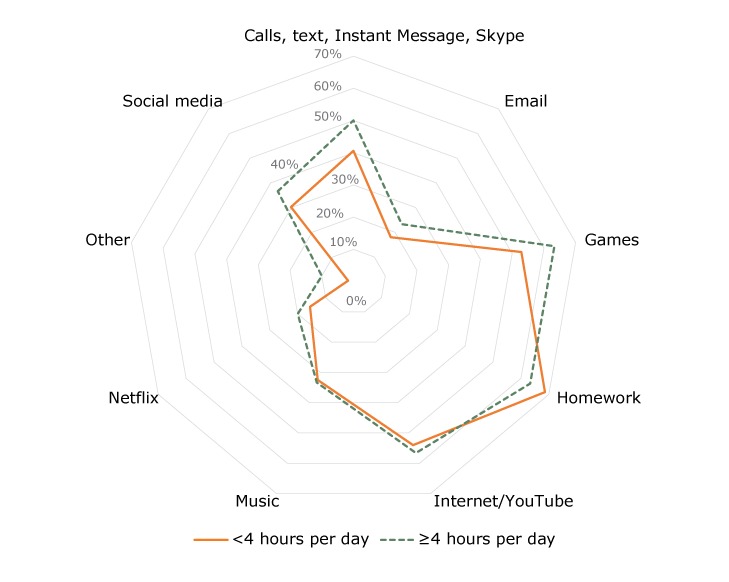
Frequency of reasons for use of smartphones, desktops, or laptops by children on weekday in study households, according to level of use in the reference child, Chaparral and Columbus, New Mexico, 2016. Parents could indicate more than 1 reasons for use; thus, percentages do not sum to 100. **Table:** 

Parents reported multiple reasons why children (as a group) in their households used desktops or laptops and smartphones on weekdays (Figure 1). Two-thirds of all users used these devices for homework. Just more than half of all users used them for games and for internet/YouTube. No single reason for use was significantly associated with heavy weekday or heavy weekend use.

Use	Level of Use	
	≥4 Hours per Day	<4 Hours per Day
Calls, text, Instant Message, Skype	50.0	40.6
Email	23.3	18.0
Games	63.3	52.9
Homework	63.3	68.6
Internet/YouTube	56.6	54.0
Music	33.3	32.5
Netflix	20.0	15.6
Other	10.0	1.7
Social media	36.6	30.2

**Figure 2:**
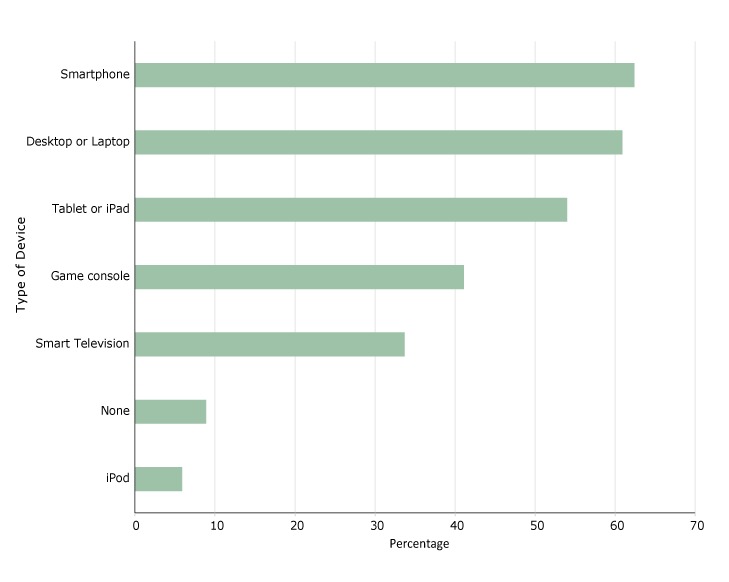
Frequency of use of types of electronic devices by children in study households, Chaparral and Columbus, New Mexico, 2016. **Table:** 

Among devices used by all children in study households, smartphones (62.4%) and desktop or laptops (60.9%) were dominant (Figure 2). Only 8.9% of children used none of the devices listed. No devices were significantly associated with heavy weekday or weekend screen time.

Type of Device	Percentage
iPod	5.9
None	8.9
Smart television	33.7
Game console	41.1
Tablet or iPad	54.0
Desktop or laptop	60.9
Smartphone	62.4

Among mothers, 89.2% used smartphones, 25.0% used desktops or laptops, and 7.3% used game consoles. Paternal patterns of use were similar. Parental patterns of use were not significant predictors of heavy screen use among children.

## Discussion

This study found that in 2 rural communities in New Mexico near the Mexico border, most families had cell phones and access to the internet in 2016. Among these families, one in 4 had a child aged 6 to 10 years who spent 2 hours or more per weekday using electronic devices at home, and one in 7 had a child who spent 4 hours or more per weekday using electronic devices at home. Most families reported that a television was on while children ate, and someone was using electronic devices during meals in one-quarter of the households. Social norms of television use during meals, not eating as a family frequently, encouraging children to entertain themselves with electronics, and not participating as a family in physical activities appear to be risk factors for heavy screen use in this study population.

In aggregate, the total screen time reported for many of these elementary-school–aged children exceed previous recommendations to limit screen time to 2 hours per day (18). Comparison with other populations of children is difficult because of differences in ages of study populations, outcome measures, and scope. In a population of Latino participants in the Special Supplemental Nutrition Program for Women, Infants, and Children (WIC) in Oregon, 42% of children aged 2 to 5 years spent 2 hours or more per day on noneducational screen time (19). The National Health and Nutrition Examination Survey found that 47% of children aged 2 to 15 years spent more than 2 hours per day viewing television and video and using computers (20). Among Hispanic media users aged 8 to 12 years in 2015, mean daily screen time was 5 hours and 34 minutes nationally (8). The National Survey of Children’s Health (NSCH) reported weekday screen time for children who were more similar in age to those in our study population. For 2011–2012, NSCH reported that 7.8% of children aged 6 to 11 years old in New Mexico watched television or videos or played video games for 4 hours or more per weekday and that 2.9% spent 4 hours or more using computers, games, and other devices per weekday (17). Even if these percentages are summed (10.7%), the prevalence of heavy weekday screen use reported by NSCH is lower than the 14.9% reported in our study. Finally, comparison with screen time among Mexican children would be of interest, but the most comparable data available for Mexico, for children and teenagers aged 10 to 14 years, show that 27.7% have an average of more than 4 hours per day of screen time (21).

Our study suggests that it is important to measure screen time on both weekdays and weekends among school-aged children and that because weekend use is greater, measuring only weekday use might substantially underestimate total use. This finding is consistent with the findings of a 2006–2007 study of television viewing among mostly Mexican American fourth-graders in low-income schools along the Texas–Mexico border, where median television viewing was greater on weekend days than on weekdays (2.5 hours vs 1.5 hours) (22). Most studies do not distinguish between weekend use and weekday use (23).

Another study conducted in the US–Mexico border region found that parental rules or norms limiting television viewing were associated with less television viewing among children (22). This finding is consistent with our finding that having a television on during meals is associated with heavy screen use on both weekdays and weekends. Our finding on television viewing during meals is also consistent with the findings of other studies showing that children in homes where the television is on all or most of the time are more likely to have more screen time than other children have (19,24). Watching television during meals is associated with poorer diets among children (25). A study in Texas found that three-quarters of urban overweight or obese Mexican American children aged 6 to 8 years had televisions in their bedrooms (26).

For weekend use, several norms in addition to television use during meals were significant in the adjusted analysis. These same associations were suggested in weekday results but lacked significance. In general, it appears that eating meals and engaging in activities as a family limits screen time, while using electronic devices to keep children occupied increases it. Examinations of such family activities in relation to screen time were reported previously (27,28). The American Academy of Pediatrics has recommended positive parenting activities, such as playing together, as one way to decrease screen time (3).

Our study population’s access to computers and internet services can be compared with such access among the Hispanic population nationally. The 2015 American Community Survey established that 68.3% of Hispanic households had desktops or laptops and 70.9% had internet service; in limited–English-speaking populations, such as the one in our study, 53.0% of households had a computer (29). In our study, 108 (53.3%) households had cell phone plans, and 186 (92.1%) had internet access.

The extent to which our study population reflects the Mexican American population living in *colonias* in New Mexico is not clear. In our study population, 68.3% of households had an annual income of less than $24,000. This percentage is comparable to the 69.1% of households of all races/ethnicities with an annual income of less than $25,000 in 2016 in Columbus, New Mexico, but it is different from the 49.5% of households with an annual income of less than $25,000 in Chaparral (12). Some aspect of how the study sample was collected might have resulted in the recruitment of families whose incomes are lower than the average income of residents in the 2 *colonias* in our study. The study’s possible inclusion of low-income families who avoid participation in the census because of their undocumented status might account for this bias.

This study has several limitations. First, the study population was a convenience sample, and selection bias might have operated in the recruitment process and/or in the choice of the reference child by the parent when more than one child was eligible. No random sampling of households was considered possible in these communities. Consequently, the reported estimates might differ from those in these communities overall or in other New Mexico *colonias*. Second, parental awareness of the more socially desirable responses to questions about use of electronic devices by children might have introduced a reporting bias toward underreporting screen time or household norms that encouraged it, such as choosing the child with less screen time as the reference child. Third, the sample size was small and may have been underpowered to detect associations between household norms and children’s screen time. The study’s strengths were the collection of data by trained, bilingual, local promotor–researchers and the 78% participation rate. To our knowledge, ours is the first assessment of total screen time, as opposed to television viewing (23), in *colonias* along the US–Mexico border.

Although some Hispanic children of Mexican heritage live in poor, remote communities in the Southwest and their families might have limited skills in English, the assumption that their access to the internet or electronic devices is limited would be incorrect. Along with adopting an American diet and its attendant risk of obesity (30), Mexican American children whom we studied in these New Mexican communities have adopted the same levels of computer use and other electronic screen use as have non-Hispanic white children elsewhere in the United States.

Checking the epidemic of obesity among the Hispanic population in the United States in such communities will depend on making behavioral changes early in life and addressing the twin issues of diet and physical inactivity, including reducing screen time without cutting off access to screen time that might be beneficial (3). Strategies found to be effective in nonminority populations in reaching such goals need to be tested in Hispanic and other racial/ethnic minority populations.

## References

[1] Hales CM , Carroll MD , Fryar CD , Ogden CL . Prevalence of obesity among adults and youth: United States, 2015–2016. NCHS data brief, no 288. Hyattsville (MD): National Center for Health Statistics; 2017. 29155689

[2] Centers for Disease Control and Prevention. High school Youth Risk Behavior Survey. New Mexico 2015 results. https://nccd.cdc.gov/youthonline/app/Results.aspx?LID=NM. Accessed December 10, 2017.

[3] Council on Communications and Media. Media use in school-aged children and adolescents. Pediatrics 2016;138(5):e20162592. 10.1542/peds.2016-2592 27940794

[4] de Jong E , Visscher TL , HiraSing RA , Heymans MW , Seidell JC , Renders CM . Association between TV viewing, computer use and overweight, determinants and competing activities of screen time in 4- to 13-year-old children. Int J Obes (Lond) 2013;37(1):47–53. 10.1038/ijo.2011.244 22158265

[5] Doherty M , Santiago-Torres M , Cui Y , Schoeller D , LaRowe T , Adams A , The association between screen time and weight status in Hispanic children. BAOJ Obes Weight Loss Manag 2015;1(1):1–12. 27747312 PMC5061453

[6] Tremblay MS , Aubert S , Barnes JD , Saunders TJ , Carson V , Latimer-Cheung AE , Sedentary behavior research network (SBRN) — Terminology Consensus Project process and outcome. Int J Behav Nutr Phys Act 2017;14(1):75. 10.1186/s12966-017-0525-8 28599680 PMC5466781

[7] Common Sense Media Inc. Fact sheet: The Common Sense census: media use by kids age zero to eight, 2017. Hispanic/Latino children’s media use. San Francisco (CA); 2017. https://www.commonsensemedia.org/sites/default/files/uploads/research/0-8census_latinomediause_release.pdf. Accessed December 21, 2017.

[8] Common Sense Media Inc. The Common Sense census: media use by tweens and teens. San Francisco (CA); 2015. https://www.commonsensemedia.org/sites/default/files/uploads/research/census_executivesummary.pdf. Accessed December 21, 2017.

[9] Green D , Peñaloza L , FitzGerald C . New Mexico Youth Risk and Resiliency Survey: middle school survey results 2015, Otero County. Epidemiology and Response Division, New Mexico Department of Health; School and Family Support Bureau, New Mexico Public Education Department; and University of New Mexico Prevention Research Center. 2016. http://www.youthrisk.org/pdf/YRRS-2015-MS-countyreport-otero.pdf. Accessed December 11, 2017.

[10] Fakhouri TH , Hughes JP , Brody DJ , Kit BK , Ogden CL . Physical activity and screen-time viewing among elementary school-aged children in the United States from 2009 to 2010. JAMA Pediatr 2013;167(3):223–9. 10.1001/2013.jamapediatrics.122 23303439

[11] Weng SF , Redsell SA , Swift JA , Yang M , Glazebrook CP . Systematic review and meta-analyses of risk factors for childhood overweight identifiable during infancy. Arch Dis Child 2012;97(12):1019–26. 10.1136/archdischild-2012-302263 23109090 PMC3512440

[12] Sharkey JR , McDonald JA , Kunz S , Umstattd Meyer R . *Salud Para Usted Y Su Familia* [Health For You And Your Family]: childhood obesity prevention in Arizona, New Mexico, and Texas border areas. Proceedings of the 48th Annual Conference of the Society for Nutrition Education and Behavior; 2015 Jul 25–28; Pittsburgh, Pennsylvania. In: J Nutr Educ Behav 2015;47(4):S97–S98; Abstract no. NP13.

[13] US Department of Housing and Urban Development. History of colonias in Arizona and New Mexico. https://www.hud.gov/states/shared/working/groups/frmwrkcoln/history. Accessed December 21, 2017.

[14] US Census Bureau. American Factfinder. https://factfinder.census.gov/faces/tableservices/jsf/pages/productview.xhtml?src=CF. Accessed December 29, 2017.

[15] Johnson CM , Sharkey JR , Dean WR , St John JA , Castillo M . Promotoras as research partners to engage health disparity communities. J Acad Nutr Diet 2013;113(5):638–42. 10.1016/j.jand.2012.11.014 23375463 PMC3633728

[16] St John JA , Johnson CM , Sharkey JR , Dean WR , Arandia G . Empowerment of promotoras as promotora-researchers in the Comidas Saludables & Gente Sana en las Colonias del Sur de Tejas (Healthy Food and Healthy People in South Texas Colonias) program. J Prim Prev 2013;34(1-2):41–57. 10.1007/s10935-013-0296-1 23404423

[17] Data Resource Center for Child & Adolescent Health. National Survey of Children’s Health 2011/12. http://childhealthdata.org/browse/survey. Accessed December 20, 2017.

[18] Khalsa AS , Kharofa R , Ollberding NJ , Bishop L , Copeland KA . Attainment of ‘5-2-1-0’ obesity recommendations in preschool-aged children. Prev Med Rep 2017;8:79–87. 10.1016/j.pmedr.2017.08.003 28856085 PMC5573793

[19] Asplund KM , Kair LR , Arain YH , Cervantes M , Oreskovic NM , Zuckerman KE . Early childhood screen time and parental attitudes toward child television viewing in a low-income Latino population attending the Special Supplemental Nutrition Program for Women, Infants, and Children. Child Obes 2015;11(5):590–9. 10.1089/chi.2015.0001 26390321 PMC4628228

[20] Sisson SB , Church TS , Martin CK , Tudor-Locke C , Smith SR , Bouchard C , Profiles of sedentary behavior in children and adolescents: the US National Health and Nutrition Examination Survey, 2001–2006. Int J Pediatr Obes 2009;4(4):353–9. 10.3109/17477160902934777 19922052 PMC2891818

[21] Encuesta Nacional de Salud y Nutricion. 2012: Resultados de actividad física y sedentarismo en personas de 10 a 69 años. https://ensanut.insp.mx/doctos/analiticos/ActividadFisica.pdf. Accessed May 2, 2018.

[22] Springer AE , Kelder SH , Barroso CS , Drenner KL , Shegog R , Ranjit N , Parental influences on television watching among children living on the Texas-Mexico border. Prev Med 2010;51(2):112–7. 10.1016/j.ypmed.2010.05.013 20561969

[23] Aftosmes-Tobio A , Ganter C , Gicevic S , Newlan S , Simon CL , Davison KK , A systematic review of media parenting in the context of childhood obesity research. BMC Public Health 2016;16(1):320. 10.1186/s12889-016-2981-5 27076213 PMC4831097

[24] Rideout VJ , Foehr UG , Roberts DF . Generation M^2^: media in the lives of 8- to 18-year-olds. Menlo Park (CA): Henry J. Kaiser Family Foundation; 2010. https://www.kff.org/other/event/generation-m2-media-in-the-lives-of/. Accessed February 5, 2018.

[25] Coon KA , Goldberg J , Rogers BL , Tucker KL . Relationships between use of television during meals and children’s food consumption patterns. Pediatrics 2001;107(1):E7. 10.1542/peds.107.1.e7 11134471

[26] Salahuddin M , Pérez A , Ranjit N , Kelder SH , Barlow SE , Pont SJ , Predictors of severe obesity in low-income, predominantly Hispanic/Latino children: the Texas Childhood Obesity Research Demonstration Study. Prev Chronic Dis 2017;14:E141. 10.5888/pcd14.170129 29283881 PMC5757383

[27] Gingold JA , Simon AE , Schoendorf KC . Excess screen time in US children: association with family rules and alternative activities. Clin Pediatr (Phila) 2014;53(1):41–50. 10.1177/0009922813498152 23922251 PMC11331274

[28] Totland TH , Knudsen MD , Paulsen MM , Bjelland M , Van’t Veer P , Brug J , Correlates of irregular family meal patterns among 11-year-old children from the Pro Children study. Food Nutr Res 2017;61(1):1339554. 10.1080/16546628.2017.1339554 28680386 PMC5492084

[29] Ryan C , Lewis JM . Computer and internet use in the United States: 2015. American Community Survey reports. Washington (DC): US Census Bureau; 2017. www.census.gov/content/dam/Census/library/publications/2017/acs/acs-37.pdf. Accessed December 10, 2017.

[30] Vera-Becerra LE , Lopez ML , Kaiser LL . Child feeding practices and overweight status among Mexican immigrant families. J Immigr Minor Health 2015;17(2):375–82. 10.1007/s10903-013-9879-4 23996642

